# Enhancing Lettuce Yield through Innovative Foliar Spray of Biopolymers Derived from Municipal Biowastes

**DOI:** 10.3390/plants13121664

**Published:** 2024-06-16

**Authors:** Ferdinando Fragalà, Erika Salvagno, Emanuele La Bella, Rossella Saccone, Elio Padoan, Enzo Montoneri, Jennifer Miccichè, Daniela Ferrarello, Andrea Baglieri, Ivana Puglisi

**Affiliations:** 1 Dipartimento di Agricoltura, Alimentazione e Ambiente, Università di Catania, 95123 Catania, Italy; ferdinando.fragala@phd.unict.it (F.F.); erika.salvagno@phd.unict.it (E.S.); emanuele.labella@phd.unict.it (E.L.B.); rossella.saccone@unict.it (R.S.); micci97@outlook.it (J.M.); daniela.ferrarello@unict.it (D.F.); ipuglisi@unict.it (I.P.); 2 Dipartimento di Scienze Agrarie, Forestali e Alimentari, Università di Torino, 10095 Grugliasco, Italy; elio.padoan@unito.it (E.P.); enzo.montoneri@gmail.com (E.M.)

**Keywords:** digestate, biostimulant, biopolymers, ozonization, MBW, *Lactuca sativa*

## Abstract

Municipal waste biomass could be valorized as an alternative feedstock to produce compounds beneficial for agricultural applications. The foliar spray application of biostimulants emerges as a promising and innovative technique due to its environmental safety and ability to enhance crop yields. In recent years, the exploitation of biopolymers obtained through alkaline hydrolysis of the solid anaerobic digestate from municipal biowastes has attracted researchers’ interest. The aim of this study is to investigate the effects on lettuce growth of a product obtained through alkaline hydrolysis from municipal biowaste, Biopolymers (BPs), and of a derivate subjected to a further oxidation process, Biopolymers Oxidate (BPs OX). The effects of the treatments at various concentrations were evaluated by monitoring plant growth and observing the trends in the activities of the main enzymes involved in the nitrogen metabolic pathway of lettuce. Results suggest that the best treatments in terms of fresh weight were achieved by using BPs at 10 mg/L and BPs OX at 100 mg/L, increasing yield by around 28% and 34%, respectively. The innovative aspect of this work was to make easier for farmers the biopolymers application by testing a foliar spray methodology for BPs and BPs OX, which has never been tested before in any crop.

## 1. Introduction

Exponential population growth, industrialization and changes in lifestyle are creating the conditions to increase environmental pollutants and production of waste [1,2]. According to estimates by the United Nations and the World Bank [3], based on estimated population increase and gross domestic product (GDP), total solid waste will increase from approximately 17 billion tons produced each year to 27 billion tons by 2050. Furthermore, the growing need to produce energy and find new renewable energy sources suggests considering vegetable biomasses as an alternative feedstock to fossils to produce biofuel and chemicals. However, growing plants dedicated to biomass production creates the problem of subtracting soil from food production [4]. One of the possible solutions may be to reuse municipal biowaste (MBW) as a raw material to produce bioenergy or bioproducts suitable for chemicals. There are two essential reasons for choosing municipal biowaste: (i) it is the only negative cost feedstock, and (ii) it is easily available in every urban settlement. The European Commission considers landfill an unsuitable treatment of solid waste due to its high costs in terms of landscape degradation and environmental pollution. The waste in landfills leaches into groundwater and produces greenhouse-effect gases such as methane, carbon dioxide and other emissions that may cause serious health problems. Conversely, recycling and recovery of waste are suitable treatments for eco-sustainable waste management [5].

MBW fractions can be successfully recycled in order to obtain a wide variety of products with different characteristics which might be used in various fields for several purposes: agriculture, biofuel production, production of energy, bioplastic production, etc. [6]. Different methods for treating biowaste have been proposed, and a key challenge for the future is an ecologic transition caused by recycling and using low-environmental-impact techniques to obtain high-value-added products to reuse. MBW may be processed by anaerobic fermentation, yielding the production of biogas, anaerobic digestate and compost. Furthermore, digestate and compost may be converted into ecofriendly and value-added biobased products for multipurpose applications in various fields, including the agricultural area [7]. The agricultural applications of these biobased products to develop new sustainable products able to improve crop yields by acting as biostimulants and soil conditioners are attracting the interest of researchers and industry. Biostimulants have an essential role in improving the growth and productivity of plants through enhancing the efficiency of absorption and assimilation of nutrients and water, and improving the plant tolerance to abiotic and biotic stress conditions [8]. Therefore, their use could make possible a huge reduction in chemicals, and this aligns with the need for a remarkable reduction in environmental pollution and greenhouse gas emissions.

Several studies have demonstrated the potential of bioproducts from various sources as growth promoters across different plant growth stages. Guilayn et al. [9] used a digestate from the anaerobic digestion plant to extract by alkaline hydrolysis a pool of dissolved organic matter containing humic-like substances (HS-like), which have shown a biostimulant effect on lettuce. Bento et al. [10] reported that humic extracts derived from hydrochar and Amazonian Dark Earth significantly enhanced maize seed germination and functioned as effective plant growth promoters. Similarly, Savarese et al. [11] investigated the biostimulant effects of potassium humate from leonardite and compost tea from green compost on the productivity and nutritional status of lettuce plants. The study also investigated the effects on the primary and secondary metabolism of the treated plants, either alone or in combination with a commercial microbial inoculum consisting mainly of arbuscular mycorrhizal fungi. The results indicated that these products have potential as tools to improve crop productivity and plant nutritional and metabolic status.

HS-like may be extracted from different sources such as leonardite, peat, compost, vermicompost, anaerobic digestate, etc. Independently from their origin, the formation and the structure of HS-like are still nowadays a matter of controversial scientific debate. Therefore, HS-like are of great interest due to the recognized importance of humic substances (HS) for understanding the fertility of soils, but also to their remarkable applications in agriculture as soil conditioners and biostimulants [9].

Furthermore, in recent years, Montoneri et al. [6] have obtained new soluble biopolymers by chemical hydrolysis (BPs) and oxidation (BPs OX) from fermented MBW and exhausted food plants, respectively. The BPs are constituted by a complex mix of molecules with molecular weight ranging from 5 to over 750 kDa. These molecules are characterized by several different organic moieties containing aliphatic and aromatic C substituted by acid and basic functional groups of different strengths, which are bonded or complexed with mineral elements of groups 1 to 4. These chemical features are inherited from the pristine biowastes. The molecules contained in the BPs are water soluble memories of the native lignocellulosic proximates present in the pristine fermented biowastes from which the BPs are derived. While this molecular complexity does not allow determination of the exact chemical structure of the BPs, these products have been proven to exhibit a wide range of properties and performances as soil fertilizers, plant growth and crop production inducers, bio-photosensitizers, oxidation catalysts, polymers for manufacturing mulch films, composite pellets, composite plastic articles and high-performance surfactants. The reason for the performance of BPs in the different sectors of agriculture and chemical industry is likely due to the fact the they contain different active principles. Yet isolation and understanding of the specific roles of the active principles have not been possible yet. This constitutes a scientific gap in the understanding of the mechanism of the multipurpose performances of BPs, although, empirically, from the practical point of view, the BPs have been proven to compete with synthetic commercial products derived from fossil sources.

In this state-of-the-art context, the BPs were proven to exert a plausible biostimulant effect on the seed germination process at concentrations ranging from 1 to 5000 mg L^−1^ in cress and lettuce but showed no effect on tomato seeds [12]. Also, Fragalà et al. [7] showed that the application of 150 kg/ha BPs to the soil allows the lettuce growth to increase, the nutrient use efficiency to be enhanced and the nitrogen lixiviation to be reduced by improving the nitrogen adsorption through the stimulation of the N metabolism and the protein accumulation, thus allowing a 40% reduction in the consumption of mineral fertilizers. The BPs OX were also tested for the seed germination of different species, showing a quite different behavior with respect to BPs as, at low concentrations (1–100 mg L^−1^), a positive effect on cress and lettuce was detected, while, at the highest concentration (5000 mg L^−1^), a phytotoxic effect was observed [12]. This phytotoxic effect was useful and tested to act as a potential fungicide on multiple targets such as *Botrytis cinerea*, *Sclerotinia sclerotiorum*, *Monilia* sp., *Sclerotium rolfsii* and *Phytophthora nicotianae* [12]. These findings revealed that efficient, ecofriendly, sustainable biobased agrochemicals and fertilizers could be obtained from municipal biowaste to replace or decrease the consumption of conventional mineral fertilizers. 

The aim of the present work was to investigate, for the first time, the biostimulant effects of BPs and BPs OX on lettuce growth. Among vegetable crops cultivated in the Mediterranean area, lettuce (*Lactuca sativa* L.) is often considered a crop that is moderately sensitive to salt which requires for its cultivation the use of biostimulants in addition to regular chemical fertilization [13]. In this respect, lettuce may represent a good plant model for the evaluation of biostimulant effects. In the present work, the BPs and BPs OX were applied by a foliar spray application methodology never tested before in any food crop with these biopolymers. The effects of the treatments at different concentrations were evaluated by monitoring the plant growth and the trend of the activities of the main enzymes involved in the nitrogen metabolic pathway of lettuce.

## 2. Results

### 2.1. Morpho-Biometric Parameters

All the experimental treatments exerted a significant effect on the morpho-biometric characteristics of the treated lettuce compared to the control (Table 1), determining their increase during cultivation with all the BPs tested at all concentrations. The only exception was the ash values, which showed no significant differences among all treatments and the control.

In the application of BPs OX, significant increases were detected in all growth parameters but lettuce length at all concentrations when used at 10 mgL^−1^. As for the use of BPs, no differences were detected in ash contents.

Overall, the best performances, mainly regarding the fresh weight of the edible part, which is the most important commercial parameter, were obtained in the treatments with BPs OX 100 and BPs 10, reaching an increase in fresh weight of around 34% and 28%, respectively.

### 2.2. Chlorophylls and Carotenoid Contents

**Figure 1:**
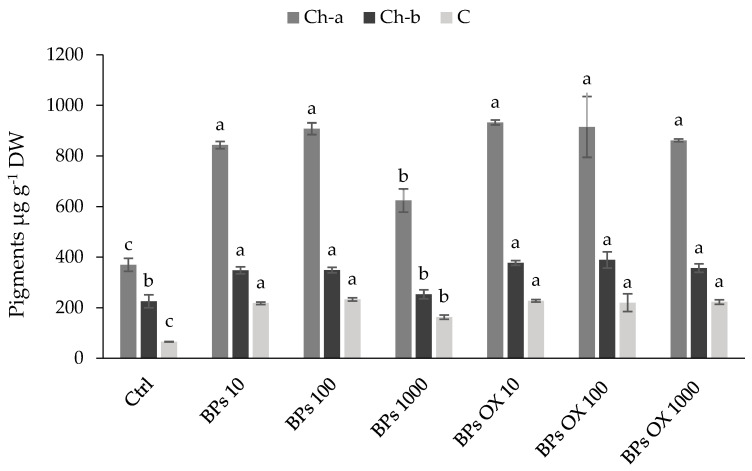
Chlorophyll-a (Ch-a), chlorophyll-b (Ch-b) and carotenoids (C) in lettuce. Error bars indicate standard deviation (±SD). Values followed by different letters are significantly different (*p* < 0.05).

In Figure 1, the contents of chlorophylls and carotenoids, measured at the end of the experiment, are reported. All treated lettuces showed a great increase in all pigments with respect to the control. In particular, all treatments enhanced chlorophyll-a, showing a percentage increase with respect to the control from 69% (BPs 1000) to 152% (BPs OX 10). Chlorophyll-b was higher than the control in all the treated lettuce, except that treated with BPs 1000, which showed a value similar to the control. The trend of carotenoids was aligned with chlorophyll-a, with a percentage increase that was always over 100% in all treated lettuce with respect to the control.

### 2.3. Protein Extraction and Enzymatic Activities

**Figure 2:**
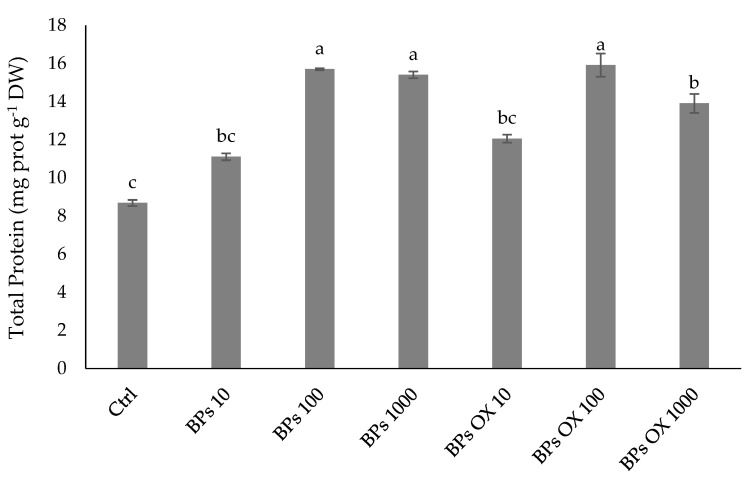
Total protein content in lettuce. Error bars indicate standard deviation (±SD). Values followed by different letters are significantly different (*p* < 0.05).

According to the morpho-biometric traits and pigments, the total protein content in lettuce subjected to all treatments was always significantly higher than that in the control (Figure 2), except for in those treated with BPs 10 and BPs OX 10, which showed values similar to the control. The best performances were obtained in the BPs OX 100, BPs 100 and BPs 1000 treatments, with an evident increase of 83%, 81% and 76% being recorded, respectively.

**Figure 3:**
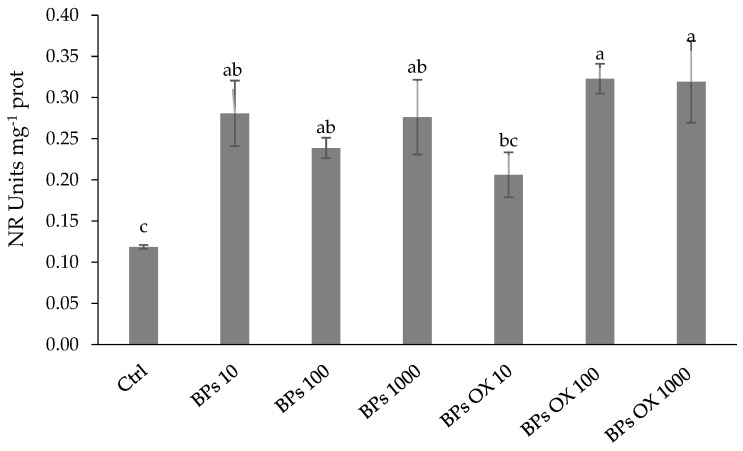
Nitrate reductase activity. Error bars indicate standard deviation (±SD). Values followed by different letters are significantly different (*p* < 0.05).

The nitrate reductase (NR) activities (Figure 3) in lettuces showed a significant increase under all the treatments tested, except for BPs OX 10, for which a value similar to the control was recorded. The highest values were registered with BPs OX 100 and BPs OX 1000, which increased the NR activity with respect to the control by around 171% and 168%, respectively.

**Figure 4:**
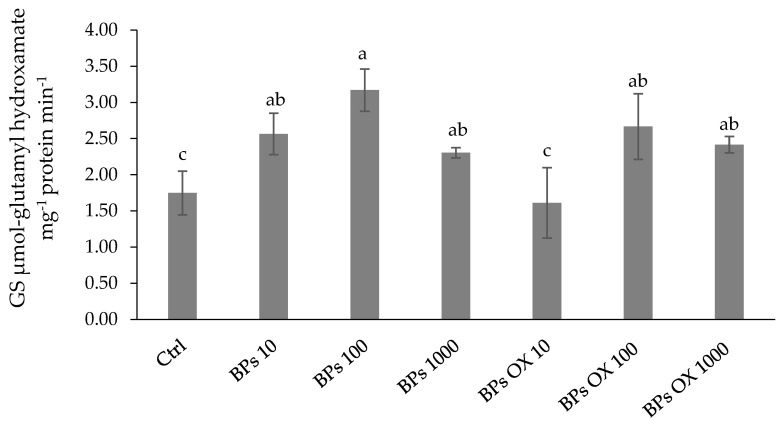
Glutamine synthetase activity. Error bars indicate standard deviation (±SD). Values followed by different letters are significantly different (*p* < 0.05).

Figure 4 shows glutamine synthetase (GS) activity measured in lettuce leaves. The activities showed a similar trend to NR, although the increases were consistently lower than those observed for NR activities. The highest GS activity was recorded with the BPs 100 treatment, which showed an increase of about 81% with respect to the control. All other treatments increased activities with respect to the control, except for BPs OX 10, which had recorded values similar to the control.

**Figure 5:**
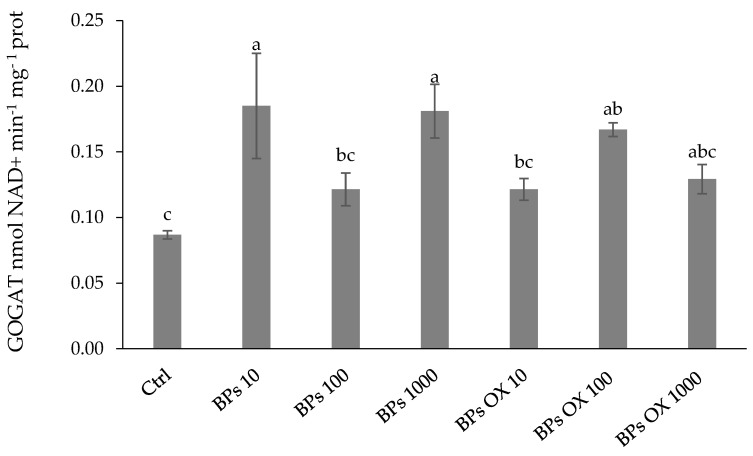
Glutamate synthase activity. Error bars indicate standard deviation (±SD). Values followed by different letters are significantly different (*p* < 0.05).

The glutamate synthase (GOGAT) activity (Figure 5) exhibited a similar performance to the previous enzymatic activities. The highest activities were recorded with the BPs 10 and BPs 1000 treatments, with an increase with respect to the control of 112% and 108%, respectively.

**Figure 6:**
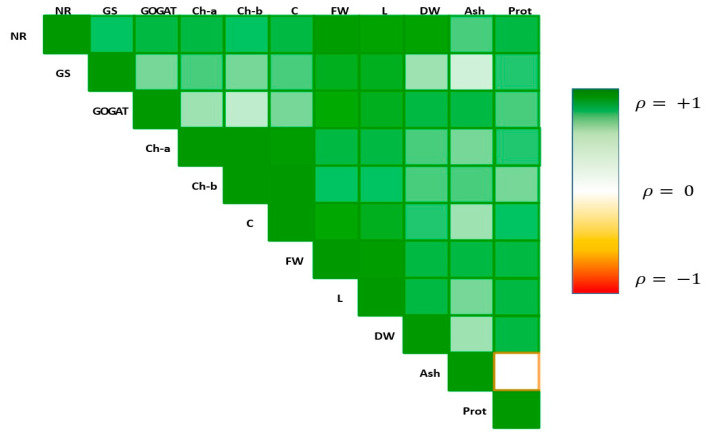
Pearson’s correlation coefficients of all the analyzed parameters in the experimental trials. Dataset: NR: nitrate reductase, GS: glutamine synthetase, GOGAT: glutamate synthase, Ch-a: chlorophyll-a, Ch-b: chlorophyll-b, C: carotenoids, FW: fresh weight, L: lettuce length, DW: dry weight, ash, Prot: total proteins. Each square is represented by a particular color shade according to its own Pearson’s correlation coefficient; a full green square represents a correlation value of +1 and a full red one a correlation value of −1.

**Figure 7:**
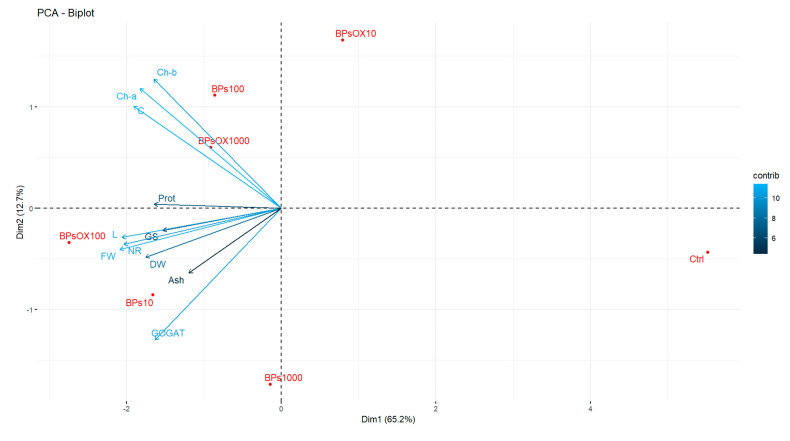
Principal component analysis (PCA) of all the analyzed parameters in the experimental trials. Dataset: Length: lettuce length, FW: fresh weight, DW: dry weight, ash, Ch-a: chlorophyll-a, Ch-b: chlorophyll-b, C: carotenoids, Prot: total proteins, NR: nitrate reductase, GS: glutamine synthetase and GOGAT: glutamate synthase.

Since all the correlation values agreed (Figure 6), a principal component analysis (PCA) was performed to assess the response of all measured parameters in the different applied treatments studied (Figure 7). The first two principal components accounted for 72.9% of the total variance in the dataset. The first component, PC1, explained 50.4%, and the second one, PC2, explained an additional 22.5% of the variance. Hence, the eigenvalues were evaluated only for PC1, and the greatest contribution in PC1 was recorded for the FW (0.35), length (0.35) and NR (0.35), followed by Ch-a (0.31).

As regards treatments, Ctrl represents the farthest from all treatments and gives us insights about the separation between the control sample and all eigenvalues. For the BPs treatments, BPs 10 is the most in the direction of the eigenvalues, followed by BPs 100. For the BPs OX treatments, BPs OX 100 is the best pointed out. In particular, BPs OX 100 is the most highly correlated with FW and NR, which are two of the three parameters with the greatest contribution of PC1.

## 3. Discussion

Prior to the present work, the biostimulant effect of BPs applied to soil in solid form was proven in different plant species [14], including also lettuce [7,12]. However, the foliar spray application of BPs in solution form had never been tested with food crops. The BPs OX, with molecular weight of about 0.2 kDa (see Section 4.1), were tested for the lettuce preliminary germination step. These proved the induction of the germination process by BPs OX at concentrations ranging between 10 and 1000 mg L^−1^ [12]. At higher BPs OX concentrations, a potential in vitro antifungal effect against multiple targets such as *Botrytis cinerea*, *Sclerotinia sclerotiorum*, *Monilia* sp., *Sclerotium rolfsii* and *Phytophthora nicotianae* was demonstrated. For BPs, the potential effect as enhancers of the lettuce seed germination process was proven in the 10–5000 mg L^−1^ concentration range. The results of the present work confirm the biostimulant effect of BPs and BPs OX in the 10–1000 mgL^−1^ range also in the lettuce post-germination process for the crop cultivation.

Fragalà et al. [7] also showed that the application to the soil of solid BPs at a rate of 50 and 150 kg/ha greatly contributed to the ecofriendly sustainability of lettuce production by reducing the consumption of mineral fertilizers during lettuce cultivation, and by the consequent expected mitigation of the environmental impact caused by the mineral nutrient leaching. Although comparing the quantity of solid BPs used by Fragalà et al. [7] and the spray application used in the present study for each plant may result in a very challenging calculation, some speculation suggests that the spray application allows the consumption of BPs to be reduced.

In broad terms, following the lettuce production guideline provided by Buturi et al. [15], in order to achieve a good growth and maximize lettuce yields, farmers are used to cultivating around 50,000–60,000 plants per hectare, with a space between rows of 27–60 cm and space between lettuces in the rows of 18–30 cm. Considering the highest value of 60.000 lettuce plants/ha, the treatments with 50 kg/ha and 150 kg/ha [7] consume 0.83 g and 2.5 g of BPs powder per plant, respectively. In the spray application adopted in the present work, each lettuce was sprayed three times until the whole surface was wet, resulting in a consumption of 0.1 L of solution per plant. Considering the highest concentration of BPs (1000 mg L^−1^), a consumption of 0.1 g per plant of solid BPs can be envisaged. Thus, it can be reasonably asserted that the spray application significantly reduces the amount of solid BPs used. This reduction, with respect to the treatment proposed by Fragalà et al. [7], applies even with respect to the lower 50 kg/ha dose.

The reported morpho-biometric traits clearly show that all the BPs treatments positively influenced the crop yield (Table 1). The best performances, mainly regarding the fresh weight of the edible part, which is the most important commercial parameter, were obtained in the BPs OX 100 and BPs 10 treatments, reaching an increase in fresh weight of around 34% and 28%, respectively (Table 1). This parameter is a very important treat, as it shows the greatest contribution in PC1 (Figure 7).

The results of the present work are consistent with the previous studies on the effect of BPs on a wide range of crops [7,14]. In particular, comparing our results with the cultivation of lettuce by Fragalà et al. [7], the application of BPs to the soil at a concentration of 150 kg/ha determined an increase of around 24% with respect to the control, lower than the value obtained in the present work by foliar spraying of BPs OX 100 and BPs 10 (34% and 28%, respectively, Table 1). In terms of solid BPs, the use of foliar-sprayed BPs 10 was calculated to correspond to the consumption of 0.05 g. This value, compared to the 2.5 g of powder used by Fragalà et al. [7] for 150 kg/ha, is 50 times lower. No comparative evaluation is possible for lettuce cultivation using BPs OX.

Generally, the results of the present work suggest that the spray application for lettuce, in addition to reducing solid BPs consumption, is a better option with respect to soil application to increase the fresh weight of the edible portion of the crop. Among application methods, the foliar spraying of plant biostimulants represents an advantage in field conditions for broad leafy vegetables as it is an application method that is easier for farmers to handle, it increases fresh and dry biomass and leaf area and enhances chlorophyll biosynthesis, determining an improved yield performance [16,17,18]. Moreover, foliar spray application is considered a promising and innovative agricultural technique as it is safe for the environment, and it does not influence soil biological and biochemical properties due to the potential addition to the soil of substances that microorganisms can also metabolize [19]. Biostimulant spray application positively affects plant growth by enhancing water uptake, root and shoot growth, tolerance to abiotic stress, protein content and activities of the enzymes involved in carbon and nitrogen metabolisms [20].

With specific references to the BPs and BPs OX tested in the present work, the biostimulant effect exerted on plants may be mainly due to the presence of humic-like substances (HS-like). Similar to the BPs, HS-like are mixtures of molecules with different molecular weight and organic moieties. The foliar spray application of HS is reported in the literature to promote bioactive effects in plants, stimulate growth and development even in the presence of in situ biotic and abiotic stresses and increase agricultural productivity [21,22,23,24,25]. Yet, as for the BPs, the mechanism for the bioactive effects of HS-like is not definitely proven yet [26]. Several fertilizers and biostimulants made from HS-like are available. These products may be used to develop state-of-the-art intelligent agricultural technologies that offer increased efficiency thanks to their flexibility and structural richness [27]. Lettuce is one of the most studied vegetable species in relation to the foliar application of HS-like as the edible part of leafy vegetables develops above the soil surface [27,28,29]. Foliar spray application of HS-like, such as fulvic acids, also protects lettuce against abiotic stresses such as salinity stress and drought stress, improving also its growth and alleviating symptoms caused by cadmium toxicity [28].

The correlation between the structure of HS and HS-like as well as their biological activity is still debated [30]. According to Piccolo [31], HS may be described as supramolecular structures formed by relatively small molecules held together by non-covalent intermolecular interactions. The outside domain consists of polar groups determining HS solubility and their biological reactivity. High contents of carboxylic acids, proteins and amino acids have been associated with auxin-like activity, whereas the hydrophobic domain, composed by aromatics and amides functional groups, has been associated with gibberellic-like activity [32]. According to the structure of HS, it may be coherent to consider the BPs as a mixture containing supramolecules at different molecular weights. In foliar spray application, how exogenous inputs may be delivered to plant cell tissue may be explained by two theories. The first one hypothesizes that beneficial molecules transfer into leaf tissues via transcuticular penetration [33]; the second one is related to the penetration through leaf stomata [34].

According to the positive effect on the morpho-biometric traits of the lettuce seedlings, all spray BPs treatments enhanced pigment production with respect to the control (Figure 1), promoting production for proper foliage growth and good green color [35]. The results obtained with the foliar application are quite different from those obtained with the solid BPs applied to soil by Fragalà et al. [7], where a rather constant chlorophyll content was observed during the lettuce cultivation. This effect is probably due to the foliar spray application, which positively affects the chlorophyll content of plants [27]. This parameter is a measure of plant overall growth as it is essential for photosynthesis, whereby plants derive energy for their development, metabolism and hormonal processes [36].

Regarding the N metabolism, the rate-limiting stage of the N assimilation pathway may be linked to the incorporation of nitrate within the cells, and the cytosolic enzyme NR may be considered the limiting factor for the growth and development of plants [37]. GS and GOGAT have also been proposed to play a key role through ammonium incorporation into organic compounds by assimilating the ammonium into the amino acid skeleton as glutamine and glutamate [38,39]. Our results suggest that the enzymatic activities related to the N metabolism support the protein accumulation using both products at medium and high concentrations, while treatments at 10 ppm of both BPs and BPs OX had values not significantly higher than the control (Figure 2). Moreover, only in the BPs OX 10 treatment were the recorded values for NR, GS and GOGAT not significantly different from those of the control (Figure 3, Figure 4 and Figure 5). These results were also observed in the PCA (Figure 7), where, among the treatments, BPs OX 10 was far from all the others and provides insights into the separation of this treatment from the others. It is likely that BPs OX requires a higher concentration to be effective as a biostimulant for lettuce. Indeed, BPs OX 100 led to very promising results. On the contrary, at the germination level, the treatment with 10 mg L^−1^ BPs OX positively affected the calculated parameters in the work by Fragalà et al. [12], while the dosage of 5000 mgL^−1^ exerted a phytotoxic effect.

The biostimulant effects observed on lettuce may be all attributed to the action of bioactive compounds, presumably made of HS-like components and a complex of supramolecules, which undergo a different degree of dissociation, exerting an auxin-like activity. Indeed, Guilayn et al. [9] extracted by alkaline reaction a pool of dissolved organic matter containing HS-like components, starting with a digestate from the anaerobic digestion plant, which have shown a biostimulant effect on lettuce cultivated in hydroponic conditions.

The results of the present work add further knowledge on the multipurpose properties of the biopolymers obtained from municipal biowastes [14]. The effect of the foliar spray on the N metabolism and the consequent enhanced lettuce growth is consistent with the results obtained using other biostimulant types, such as microalgae-based extracts [40,41,42], plant-based preparations [43] and amino-acid-based biostimulants [44,45].

## 4. Materials and Methods

### 4.1. Materials

The soluble products were prepared as described in detail by Montoneri et al. [6]. Briefly, the digestate, obtained by anaerobic digestion of MBW in the Acea muncipal biowaste treatment plant (Pinerolo, TO, Italy), was hydrolyzed in water at pH 13 and 60 °C. The liquid hydrolyzate was then subjected to sedimentation, followed by centrifugation and ultrafiltration through a 5 kDa cutoff polysulphone membrane. The membrane retentate was dried at 60 °C to obtain the BPs with molecular weight greater than 5 kDa. The BPs OX were obtained by dissolving the BPs in pH 10 water and oxidizing them by bubbling a 4% ozone–oxygen stream through the aqueous solution for 64 h. The final alkaline solution was filtered through a 0.2 kDa polysulphone membrane, and the membrane retentate finally dried at 60 °C.

### 4.2. Experimental Site and Plant Material

The experimental trial was carried out in a greenhouse from June 2022 to July 2022 in a nursery located in Piazza Armerina (Enna, Italy), 400 m altitude. The climate of the area is Mediterranean, with mild winters and hot, dry summers. Seedlings of lettuce (*Lactuca sativa* L.), cultivar ‘Romana’, were provided by the local nursery and were transplanted at the stage of four true leaves into 1 L plastic pots (11 cm height, 14 cm width) filled with a standard substrate used in horticulture, Floradur^®^ bio-substrate (coarse structure, pH 6.3, EC 0.36 dS/m, salt content 1.2 g/L). Each pot, containing 1 plant, was arranged in a completely randomized design composed of three replicates for each treatment, and each replica was made of 10 seedlings. The seedlings were regularly grown according to the nursery protocols and were opportunely irrigated. Lettuces were harvested after 40 days of growth.

### 4.3. Treatments

The treatments with the two preparations, BPs and BPs OX, consisted of three consecutive independent fortnightly foliar spray applications at three different concentrations (10, 100, 1000 mg L^−1^), chosen by taking into account the results for lettuce seed previously obtained by Fragalà et al. [12]. All the treatments are listed in Table 2. Each lettuce was sprayed until the whole surface was wet (in the first treatment, 15 mL per plant; in the second treatment, 35 mL per plant; and, in the third treatment, 50 mL per plant). Control plants (Ctrl) were regularly grown without any treatment. Pots with lettuces were transferred to the laboratory, where plants were manually harvested to avoid any damage to the leaves, and, after the morpho-biometric parameters were collected, the lettuces were flash-frozen with liquid nitrogen and stored at −80° C for further enzymatic analysis.

### 4.4. Morpho-Biometric Parameters

Lettuce lengths were measured with a flexible ruler to the nearest 0.5 mm, and the edible part was separately weighed in order to obtain the fresh weight (FW). The dry weight of lettuce (DW) was obtained by placing tissues in a drying oven (Thermo Scientific Heratherm, Waltham, MA, USA) at 105 °C until a constant weight was reached then allowing them to cool inside a closed bell jar for 2 h, and, finally, the dry weights were obtained. The ashes were determined at 505 °C in a muffle (Lab 1200C Muffle Alumina Ceramic Fiber Furnace, Livingston, MT, USA). All measurements were performed on 3 plants for treatment and replicates.

### 4.5. Chlorophyll and Carotenoid Contents

The determination of the pigment contents was performed following the method proposed by Sumanta et al. [46]. For each plant, 0.5 g of fresh leaf tissue was accurately weighted, ground in a mortar with liquid nitrogen and homogenized with 10 mL of 80% acetone as extractant solvent. Homogenized sample mixture was centrifuged at 10,000 rpm for 15 min at 4 °C (Neya 16 R, Vasai, India), and 0.5 mL of supernatant was collected and mixed with 4.5 mL of the solvent. The solution mixture was analyzed for chlorophyll-a, chlorophyll-b and carotenoids content in a spectrophotometer (Jasco V-530 UV–vis spectrophotometer, Tokyo, Japan). The relative amounts of chlorophyll-a (Ch-a), chlorophyll-b (Ch-b) and carotenoids (C) were calculated as follows and expressed as mg/g leaf dry weight (DW):

Ch-a = 12.25 A663.2 − 2.79 A646.8

Ch-b = 21.5 A646.8 − 5.1 A663.2

C = (1000 A470 − 1.82 Ch-a − 85.02 Ch-b)/198

### 4.6. Protein Extraction and Enzymatic Activities

Total protein extraction from fresh lettuce tissues was performed according to the method of La Bella et al. [40]. In brief, 1 g of lettuce leaves was homogenized using an extraction buffer (1:1.25 *w*/*v* ratio) containing 220 mM mannitol, 70 mM sucrose, 1 mM EGTA, 10 mM cysteine and 5 mM HEPES–KOH pH 7.5. Samples were filtered and centrifuged at 13,000 rpm for 30 min at 4 °C (Neya 16 R, Vasai, India), and the supernatant was recovered in order to determine the total protein content by the Bradford method using 10 µL of enzyme extract. Bovine Serum Albumine (BSA) was used as a standard curve and expressed as mg protein g^−1^ DW. All measurements were performed in triplicate for treatment and replicates.

The nitrate reductase (NR) activity from the fresh protein extracts was measured according to the method of Kaiser et al. [47]. Concisely, the assay solution composed by 100 mM KH_2_PO_4_ and 100 mM KNO_3_ was incubated at 28 °C for 15 min (ICN 16 Argolab, Carpi, Italy) with 100 µL of enzyme extract. The mixture was then centrifuged at 500 rpm for 5 min, and the recovered supernatant was spectrophotometrically measured at 540 nm (Jasco V-530 UV–vis spectrophotometer, Tokyo, Japan). The activity was expressed as Unit mg^−1^ protein using a calibration curve with known concentrations of NaNO_2_.

The glutamine synthetase (GS) activity was analyzed following the method of Canovas et al. [48]. The assay was carried out in a total volume of 750 µL composed by 90 mM imidazole-HCl (pH 7.0), 60 mM hydroxylamine (neutralized), 20 mM KAsO_4_, 3 mM MnCl_2_, 0.4 mM ADP, 120 mM glutamine and 100 µL of enzyme extract. The reaction mixture was incubated for 15 min at 37 °C (ICN 16 Argolab, Carpi, Italy), then 250 µL of a mixture (1:1:1) of 10% (*w*/*v*) FeCl_3_ 6H_2_O in 0.2 M HCl, 24% (*w*/*v*) trichloroacetic acid and 50% (*w*/*v*) HCl was added to stop the reaction. The γ-glutamyl hydroxamate produced in the leaf extract was spectrophotometrically determined at 540 nm (Jasco V-530 UV–vis spectrophotometer, Tokyo, Japan) and was expressed as mol γ-glutamyl hydroxamate mg^−1^ protein min^−1^ using a standard curve of γ-glutamyl hydroxamate.

Glutamate synthase (GOGAT) assay was performed according to Avila et al. [49]. Briefly, in the assay, 25 mM Hepes–NaOH (pH 7.5), 2 mM L-glutamine, 1 mM.α-ketoglutaric acid, 0.1 mM NADH, 1 mM Na_2_EDTA and 100 µL of enzyme extract were mixed and measured by spectrophotometer (Jasco V-530 UV–vis spectrophotometer, Tokyo, Japan) following NADH oxidation at 340 nm. GOGAT activity was expressed as nmol NAD^+^ min^−1^, mg^−1^ protein using a molar extinction coefficient of 6220 L mol^−1^ cm^−1^.

### 4.7. Statistical Analyses

Data were analyzed by one-way ANOVA (*p* < 0.05) followed by Fisher’s test for multiple comparison procedures using statistical software (Minitab^®^ 19) to investigate the effect of each treatment on plants. Pearson’s correlation coefficients were calculated using GeoGebra^®^ software 6.0.741 integrating “Spreadsheet view”, “CAS view” and “Graphics view”. Principal component analysis (PCA) was performed using functions embedded in the “factoextra” R package. The “prcomp” function was set on the “scale=true” option in order to normalize the parameters. The results were displayed by using the “fviz_pca_biplot” function. Values used for Pearson’s correlations and PCA were the means of all replicates as the statistical analysis showed a *p* < 0.05.

## 5. Conclusions

The foliar spray application of biostimulants in agriculture practices is to be considered an encouraging and innovative technique as it is safe for the environment and potentially increases yields in crop production. Our results have shown new perspectives about the spray application of BPs and BPs OX used as biostimulants; it is able to enhance lettuce growth by stimulating chlorophyll accumulation and nitrogen metabolism. Although the foliar spray application of BPs deserves further investigation, our results are very promising. Taking all the results together, the best outcomes seem to be achieved by using BPs 10 and BPs OX 100. Upon considering performance and production cost, the BPs with molecular weight > 5 kDa seem a more promising and sustainable product compared to the low-molecular-weight BPs OX obtained from BPs by an additional step of oxidation and size humification. Yet BPs OX are reported to be powerful plant protective agents. New biobased agrochemicals formulations containing BPs or BPs OX may be winning ecofriendly products for the agro-chemical market to support the sustainable exploitation of biowaste materials in the current context of a circular economy.

## Figures and Tables

**Table 1:** Morpho-biometric parameters (lettuce length, FW: fresh weight, DW: dry weight, ash) of lettuces at the end of the experimental period.

	Lettuce Length (cm)	FW (g)	DW (g)	Ash (g)
Ctrl	17.33 ± 0.33 ^c^	165.33 ± 0.85 ^e^	11.56 ± 0.13 ^d^	0.92 ± 0.012 ^a^
BPs 10	23.83 ± 0.93 ^a^	212.44 ± 10.04 ^ab^	13.51 ± 0.79 ^bc^	0.93 ± 0.005 ^a^
BPs 100	23.00 ± 0.76 ^ab^	202.85 ± 3.28 ^bc^	12.93 ± 0.003 ^c^	0.93 ± 0.003 ^a^
BPs 1000	22.33 ± 0.83 ^ab^	197.03 ± 4.89 ^bcd^	14.73 ± 0.28 ^a^	0.92 ± 0.008 ^a^
BPs OX 10	20.16 ± 1.42 ^bc^	183.65 ± 2.86 ^d^	13.43 ± 0.10 ^bc^	0.93 ± 0.002 ^a^
BPs OX 100	22.66 ± 0.93 ^ab^	220.78 ± 3.39 ^a^	14.91 ± 0.61 ^a^	0.92 ± 0.010 ^a^
BPs OX 1000	22.33 ± 1.45 ^ab^	195.15 ± 5.97 ^cd^	14.60 ± 0.13 ^ab^	0.92 ± 0.011 ^a^

All data are expressed as mean ± standard error of the mean (SEM). Different letters within each column indicate significant differences according to Fisher’s protected LSD test (*p* < 0.05); ± indicates the standard error mean.

**Table 2:** Treatments performed on lettuce.

Name	Chemical Treatment of Digestate	Amount (mg L^−1^)	Foliar Application (Number)
Ctrl	-	-	-
BPs 10	Alkaline hydrolysis	10	3
BPs 100	Alkaline hydrolysis	100	3
BPs 1000	Alkaline hydrolysis	1000	3
BPs OX 10	Alkaline hydrolysis + ozone oxidation	10	3
BPs OX 100	Alkaline hydrolysis + ozone oxidation	100	3
BPs OX 1000	Alkaline hydrolysis + ozone oxidation	1000	3

## Data Availability

Data are contained within the article.
